# Research and Remedies

**Published:** 1912-08-03

**Authors:** 


### RESEARCH AND REMEDIES.

### The Dangers of Bismuth Paste.

About four or five years ago the filling of old chronic tuberculous sinuses with bismuth paste was advocated and widely practised, and so far as the closure of the sinuses was concerned there is no doubt that good results were obtained. But after a short time reports came to hand of serious symptoms due to absorption of bismuth, and several deaths were attributed to this cause. Dr. Blanchard, of Chicago, one of the earliest and most fervent advocates of the bismuth-paste treatment, now pronounces that these disadvantages are such that further usage is unjustifiable. He has conducted many experiments with a view to replacing it by something which will produce the same result without any risk of causing toxic symptoms. The ideal paste, he says in the *Medical Record*, must be non-toxic and absorbable. It must sufficiently solidify at body temperature to crowd out pus, compress unhealthy granulations, and exclude air. A formula which meets every requirement and is quite innocuous is: White wax, one part; vaseline, eight parts—mixed while boiling. If a skiagram is required to show the ramifications of the sinus he uses: Iron subcarbonate one part, white vaseline two parts. He lays stress also on the necessity for removing all sequestra before attempting this treatment, and for treating only very old-standing chronic cases in which discharge is slight and watery.

### Serum Treatment of Hyperemesis Gravidarum.

A short time ago a *résumé* was published in these columns (The Hospital, February 10, p. 494) of the various treatments which are most in favour with physicians for the condition known as the pernicious vomiting of pregnancy or as hyperemesis gravidarum. The most recent suggestion for curing really serious cases of this condition comes from France, and is published in *Les Annales de Gynécologie et d'Obstetrique* for March last. It consists in the injection into the patient of blood serum taken from a healthy woman in the second or third month of pregnancy. The theoretical basis of this treatment is that in normal pregnancy a formation of antibodies is postulated, which neutralise or destroy toxins or other detrimental substances manufactured by the fœtus; and it is further assumed that in exceptional instances this protective mechanism fails and hyperemesis ensues. Hence the injection of serum which is thought to contain the antibodies in normal amount. It would appear that three cases have been treated by French physicians in this way with striking success; but it is, of course, not claimed on the strength of this that any final verdict can be passed. The difficulty of obtaining the serum must always be a formidable one, even if further experience shows the method to be as valuable as its originators hope for.

### The Connections between Injury and Disease.

A thoughtful essay on the connections between injury and disease is contributed to the *Practitioner* by Dr. A. J. Hall, whose philosophical discussion of cause and effect should be studied not only by doctors called upon for evidence in compensation cases, but also by the lawyers representing the litigants. He points out that few deaths are the result of any one cause, illustrating this point by asking who causes the death of a criminal hanged for murder—the judge, the jury, the hangman, the prosecuting counsel, or the detectives? So also in pulmonary embolism following fracture of a bone: is "the cause" the clot of blood, the antecedent thrombosis, the micro-organisms which set up the thrombosis, the fall which caused the fracture, or the dissipated life which may have induced either the fall itself or the lack of resistance to infection? He proceeds to deal specifically with instances of particular diseases. Thus meningitis is not infrequently observed after a fall or other head injury. Dr. Hall finds it often impossible to decide definitely whether the injury has set free bacilli from a pre-existing limited focus (*e.g.*, of tubercle) or whether the fall has been merely the initial symptom of a just-established meningitis. With influenza and pneumonia the same difficulty may arise. Traumatic pneumonia does, no doubt, occur; but, on the other hand, a fall is (rarely) the first sign of onset of an acute influenzal or pneumococcic invasion. New growths, again, present many baffling problems; for the onset of the symptoms which they cause is gradual, and hence their connection with previous injury must always be a matter largely of opinion. Many other diseases are cited, and the whole paper will repay study by those engaged in accident insurance and compensation work.

### The Dangers of Camphor Antiseptic.

Attention was recently called in these columns to the dangers of using menthol as a nasal instillation in the case of infants and young children. It would now appear from an article in *La Province Médical* by Professor Maurice Perrin that similar dangers attend the use of camphor. Perrin's case was that of an infant nine weeks old suffering from slight coryza, for which the mother on the advice of a chemist introduced a small quantity of camphor and vaseline ointment into one nostril. The child at once became violently excited, grew pale, and then fell into a state of collapse from which the author succeeded in arousing it only with the greatest difficulty. There can be no doubt that the camphor alone was responsible for this, and since camphor and menthol possess very analogous properties the fact is not to be wondered at. It is probable that the ill-effects produced were the result of giving the camphor mixed with vaseline, for it has been shown that menthol is more irritating thus than dissolved in petroleum, whereas when mixed with olive oil it is said to lose its irritating properties altogether. Camphor has been much used by rhinologists on the Continent as a nasal disinfectant and hitherto no ill-results have been published. It cannot, in view of Perrin's case, be held to have no danger, and in young children and infants it should be used with caution.

### The Quantitative Estimation of Glycosuria.

Probably every general practitioner has had to face the trouble of a daily estimation of the amount of sugar present in the urine of a diabetic patient. Those who possess a polarimeter can get their results very quickly, though the accuracy of this machine is not quite perfect. But those who rely on volumetric analysis are confronted with a long and messy operation, which (if Fehling's method is used) is often unsatisfactory owing to the difficulty of deciding when the blue colour of the solution is completely discharged. Nor are the various modifications of the process which have been suggested by various authors any great improvement on the classical Fehling method. In the *Glasgow Medical Journal* Mr. H. H. Green draws attention to the "citron" method, which is used on the Continent but is almost unknown in Britain. 10 c.c. of the urine are diluted with 10 c.c. of water, if of specific gravity under 1,030; with 40 c.c. of water, if over 1,030 and under 1,040; and with 90 c.c. of water, if over 1,040. Then 20 c.c. of Fehling's solution are pipetted into an Erlenmeyer flask of 200 c.c. capacity, 10 c.c of water are added, and the mixture boiled over a Bunsen flame. While boiling 5 c.c. of the diluted urine are run in and the mixture boiled for a further two minutes. The flask is then removed, and to it are added 20 c.c. of a 20 per cent. solution of potassium iodide, 20 c.c. of 20 per cent. sulphuric acid, and 1 c.c. of starch solution (as indicator). Decinormal sodium thiosulphate is then run in from a burette until the blue colour of the starch-iodide disappears and the liquid becomes quite white. The quantity of thiosulphate solution used is to be subtracted from that required in a trial determination when no urine is added. The difference thus found is then looked up in a table which shows the mass of sugar to which it corresponds. This, therefore, enables one to say how much sugar exists in 5 c.c. of (diluted) urine, from which the actual percentage in undiluted urine is easily calculated.

